# Automated COVID-19 and Heart Failure Detection Using DNA Pattern Technique with Cough Sounds

**DOI:** 10.3390/diagnostics11111962

**Published:** 2021-10-22

**Authors:** Mehmet Ali Kobat, Tarik Kivrak, Prabal Datta Barua, Turker Tuncer, Sengul Dogan, Ru-San Tan, Edward J. Ciaccio, U. Rajendra Acharya

**Affiliations:** 1 Department of Cardiology, Firat University Hospital, Firat University, Elazig 23119, Turkey; mkobat@firat.edu.tr (M.A.K.); tarikkivrak@firat.edu.tr (T.K.); 2 School of Management & Enterprise, University of Southern Queensland, Toowoomba, QLD 4350, Australia; Prabal.Barua@usq.edu.au; 3 Faculty of Engineering and Information Technology, University of Technology Sydney, Sydney, NSW 2007, Australia; 4 Cogninet Brain Team, Cogninet Australia, Sydney, NSW 2010, Australia; 5 Department of Digital Forensics Engineering, College of Technology, Firat University, Elazig 23119, Turkey; turkertuncer@firat.edu.tr (T.T.); sdogan@firat.edu.tr (S.D.); 6 Department of Cardiology, National Heart Centre Singapore, Singapore 169609, Singapore; tan.ru.san@singhealth.com.sg; 7 Department of Cardiology, Duke-NUS Graduate Medical School, Singapore 169857, Singapore; 8 Department of Medicine, Celiac Disease Center, Columbia University Irving Medical Center, New York, NY 10032, USA; ciaccio@columbia.edu; 9 Department of Electronics and Computer Engineering, Ngee Ann Polytechnic, Singapore 599489, Singapore; 10 Department of Biomedical Engineering, School of Science and Technology, SUSS University, Clementi 599494, Singapore; 11 Department of Biomedical Informatics and Medical Engineering, Asia University, Taichung 41354, Taiwan

**Keywords:** COVID-19, heart failure, cough sounds, DNA pattern, advanced sound processing

## Abstract

COVID-19 and heart failure (HF) are common disorders and although they share some similar symptoms, they require different treatments. Accurate diagnosis of these disorders is crucial for disease management, including patient isolation to curb infection spread of COVID-19. In this work, we aim to develop a computer-aided diagnostic system that can accurately differentiate these three classes (normal, COVID-19 and HF) using cough sounds. A novel handcrafted model was used to classify COVID-19 vs. healthy (Case 1), HF vs. healthy (Case 2) and COVID-19 vs. HF vs. healthy (Case 3) automatically using deoxyribonucleic acid (DNA) patterns. The model was developed using the cough sounds collected from 241 COVID-19 patients, 244 HF patients, and 247 healthy subjects using a hand phone. To the best our knowledge, this is the first work to automatically classify healthy subjects, HF and COVID-19 patients using cough sounds signals. Our proposed model comprises a graph-based local feature generator (DNA pattern), an iterative maximum relevance minimum redundancy (ImRMR) iterative feature selector, with classification using the k-nearest neighbor classifier. Our proposed model attained an accuracy of 100.0%, 99.38%, and 99.49% for Case 1, Case 2, and Case 3, respectively. The developed system is completely automated and economical, and can be utilized to accurately detect COVID-19 versus HF using cough sounds.

## 1. Introduction

The COVID-19 pandemic is continuing to the present time despite recent vaccination efforts. Experts advise people to continue to wear masks, implement sanitization procedures, and avoid crowds [1,2]. Curfews still exist in many countries. COVID-19 has disrupted normal life and has strained national health resources, even more so at the beginning of the pandemic [3]. A new normal is necessary to limit its spread [4] and people are often living in isolation according to quarantine rules [5,6]. Many patients with pre-existing chronic illnesses such as heart failure (HF) suffer restricted access to routine medical care, and may thus risk acute clinical deterioration that requires hospitalization [7]. COVID-19 and HF share similar clinical presentations, such as symptoms of breathlessness and a cough; however the treatment mode is much different. Accurate differentiation of these disorders is therefore crucial for appropriate medical management, including deciding whether to promptly isolate a suspected COVID-19 patient to curb the spread of infection. Machine learning models can potentially be used to aid medical personnel in clinics and hospital settings to diagnose and triage both conditions automatically [8].

Many machine learning techniques have been reported for computer-aided diagnosis of diverse diseases [9,10,11,12] that may reduce clinician burden [13,14]. Moreover, many machine learning techniques have been used in many different disciplines [15,16,17]. In this study, a machine learning method was proposed for automatic differentiation of COVID-19 vs. HF conditions based on cough sounds, which can readily be recorded at low-cost using mobile phone technology. Details of the proposed method are elucidated in the relevant subsections.

Our group has previously described a rapid and accurate machine learning technique for the automated classification of heart valve disorders. It employed a distinctive graph pattern to generate features from heart sounds recorded on phonocardiography [18]. For the current study, we again exploit graph theory by using the chemical structures nucleotide basic units of the deoxyribonucleic acid (DNA) molecule, hence the name “DNA pattern”, for local feature (microstructure) generation in our proposed model. As both COVID-19 and HF can present with cough symptoms, we chose to study mobile phone recordings of cough sounds, which were then processed into one-second segments. The presented DNA pattern extracted 1024 features from each sound segment. The most valuable features were selected using iterative maximum relevance minimum redundancy (ImRMR) and classification was performed using the standard k-nearest neighbor (kNN) classifier [19]. We aimed to study the feasibility of feature generation when utilizing these DNA patterns, as well as the diagnostic performance of the DNA pattern-based model, for automatic classification of cough sounds for COVID-19 and HF diagnosis.

The novel aspects of the proposed model include:

- New local feature generator based on graph theory and the chemical structure of nucleotide basic units of the DNA molecule, which we labelled as DNA pattern-based.
- New prospectively acquired dataset comprising cough sounds recorded from healthy subjects, COVID-19, and HF patients using basic smart phone microphones, which we divided into standardized one-second sound segments for analysis.
- To the best our knowledge, this is the first work to automatically classify healthy subjects, HF and COVID-19 patients using cough sounds signals.
- The major contributions of this study include:
- Three distinct clinically relevant classification problems were defined: Case 1, COVID-19 vs. healthy; Case 2, HF vs. healthy; and Case 3, COVID-19 vs. HF vs. healthy.
- The DNA pattern- and ImRMR-based model combined with the standard kNN classifier attained excellent results, with greater than 99% accuracy for every Case.

Here, we review selected publications on computer-aided diagnostic systems for HF and COVID-19 detection using biomedical signals and imaging readouts, respectively. Masetic and Subasi [20] developed an electrocardiogram (ECG) method based on the autoregressive Burg method and random forest classifier, tested it on the MIT BIH arrhythmia [21], PTB diagnostic ECG [22] and BIDMC-congestive HF datasets [21,23], and reported a 100.0% accuracy rate for HF diagnosis. Tripathy et al. [24] processed ECGs from the MIT BIH arrhythmia [25] and BIDMC congestive HF datasets [21,23] using a high-pass filter and applied Stockwell-transform for time-frequency analysis to extract entropy features. Using hybrid classifiers with mean metric, 98.78% accuracy rate was reported for congestive HF detection. Porumb et al. [26] developed a convolutional neural network (CNN) model to diagnose congestive HF on single raw ECG heartbeats, and reported 100.0% accuracy after analyzing 490,505 individual ECG heartbeat signals. Abbas et al. [27] tested a DeTraC (Decompose, Transfer and Compose) CNN model on a combined chest X-ray image dataset [28,29], and reported a 93.10% accuracy rate for COVID-19 diagnosis. Jaiswal et al. [30] used a DenseNet201-based image classification model to analyze computed tomographic (CT) chest images [31], and attained a 96.25% accuracy rate for discriminating between COVID-19 (+) vs. COVID-19 (−) status. Singh et al. [32] applied a CNN model on CT chest images and attained a 93.50% accuracy rate for a binary classification of images into infected (+) vs. infected (−). Horry et al. [33] used a transfer learning-based method that analyzed X-ray, CT, and ultrasound images from four different datasets—COVID-19 image data collection [34], NIH chest X-Ray [35], Covid-CT [36], and POCOVID [37]—and for each imaging modality, calculated the performance metrics of the different analysis models that included VGG16 [38], VGG19 [38], Xception [39], InceptionResNetV2 [40], InceptionV3 [41], NASNetLarge [42], DenseNet121 [43], and ResNet50V2 [44]. For instance, F1-score values for VGG19 were 87.00%, 99.00%, and 78.00% for X-ray, ultrasound, and CT, respectively. Zebin and Rezvy [45] applied a CNN method to analyze chest X-ray images for initial COVID-19 classification into COVID-19, normal and pneumonia classes, as well as for monitoring of disease progression. They reported 90.00%, 96.80%, and 94.30% accuracy rates for VGG-16, EfficientNetB0 [46] and ResNet50 models, respectively.

## 2. Material and Method

### 2.1. Material

Using various mobile phones, cough sounds were recorded from 247 healthy subjects as well as 241 COVID-19 and 244 HF patients who attended Firat University Hospital, and stored in m4a (719), mp3 (3) or ogg (10) formats. Ethical approval for the study was obtained from the Firat University Ethics Committee. These recordings were of different durations and had to be subdivided into standardized one-second sound segments for analysis. There were 696 (32%), 906 (42%) and 554 (26%) sound segments from healthy subjects, COVID-19 and HF patients, respectively, out of a total of 2156 segments.

### 2.2. Method

**Figure 1:**
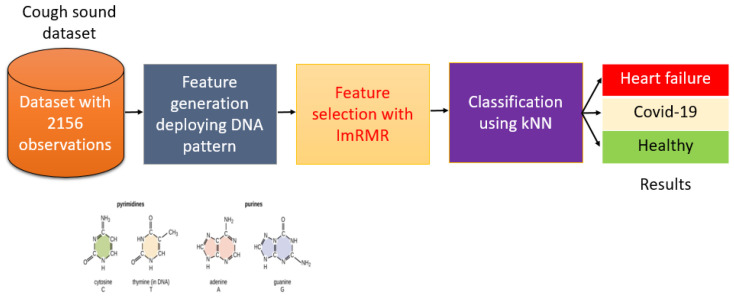
Illustration of proposed system for COVID-19 and HF detection using cough sounds.

The model comprised a graph-based local feature generator, an iterative feature selector, and classification components. The former used graphical depictions of the chemical structures of nucleotide basic units of the DNA molecule, purine and pyrimidine, to generate features from cough sounds. The optimal number of features was selected using ImRMR and classification of the chosen features performed using standard kNN classifier. A schematic of this model is shown in Figure 1.

The pseudocode of the model is given in Algorithm 1.
**Algorithm 1.** Proposed algorithm cough sound-based automatic COVID-19 and HF detection**Input:** Cough dataset (CD) with a size of 2156 × 44,100 (2156 is the total number of observations and 44,100 is the length of each observation. The sampling rate of the sound signal is 44.1 kHz), labels (y) with a length of 2156. **Output:** Results01: **for** c = 1 to 2156 **do** 02: Read each cough sound. 03: Extract 1024 features deploying DNA patterns. 04: **end for c** 05: Apply ImRMR to features generated. 06: Classify the features selected using kNN. 07: Obtain results.

#### 2.2.1. DNA Pattern

**Figure 2:**
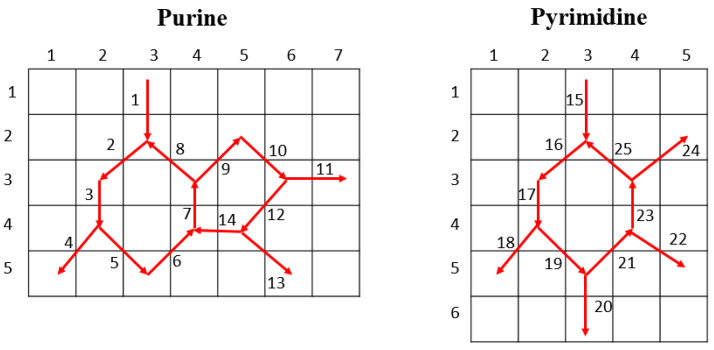
Directed cyclic graphical representations of purine (fused six- and five-membered ring conformation) and pyrimidine (six-membered ring). Individual directed paths are constructed using red arrows, which are enumerated. The initial and final points of each arrow represent the first and second parameters of the signum function for bit generation, respectively. With both structures combined, 25 bits (total number of directed paths) can be generated using 5 × 7 and 6 × 5 sized matrices (see text).

A new DNA pattern-based local feature generator was proposed. There have been several graph-based feature extraction models in the literature [18,47] and molecular structure graphs used in deep learning models and graph networks have attained high classification performance [48,49]. In this study, we used the aromatic heterocyclic chemical structures of nucleotide basic units of the DNA molecule purine with its fused six- and five-membered ring conformation; and pyrimidine, its six-membered ring to generate features from cough sound signal segments. Each purine nucleotide unit (adenine, guanine) on one DNA strand is hydrogen-bonded to the corresponding pyrimidine nucleotide unit (thymine, cytosine) of the second DNA strand (base pairing) to collectively form the DNA double helix, which is the basis of our genetic code. The chemical structures of purines and pyrimidines are topologically distinctive and can be represented as directed cyclic graphs (Figure 2). These graphs are utilized as the pattern of a histogram-based local feature generator. As can be seen in Figure 2, there are 25 edges in these two graphs, and these edges are denoted parameters of generated binary features.

**Figure 3:**
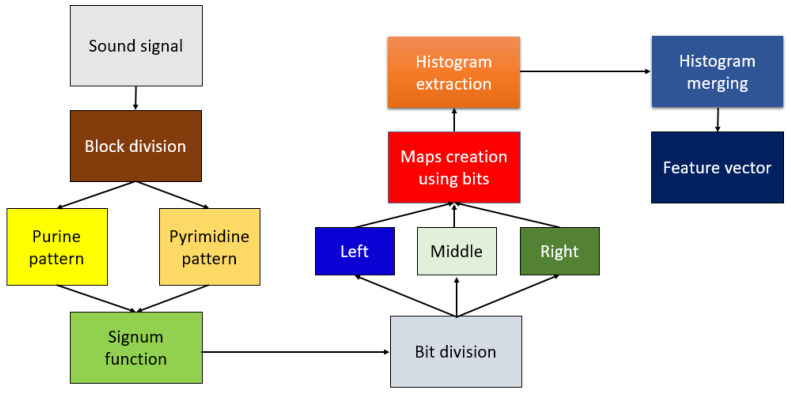
Steps involved in the generation of features using proposed DNA patterns.

A schematic of the proposed DNA pattern-based feature generation is shown in Figure 3.

Steps of the proposed DNA pattern-based feature generation:

Step 1: Divide cough sound into overlapping blocks with a size of 35.

Step 2: Create first matrix with a size of 5 × 7 using vector to matrix transformation.

Step 3: Use the purine pattern and signum function to generate 14 bits. The definition of the signum function is given in Equation (1).

(1) $$\gamma \left(f,s\right)=\{\begin{array}{c} 0,f-s<0\\ 1,f-s\geq 0 \end{array}$$

where γ(.,.), f and s are the signum function first and second parameters, respectively.

Step 4: Divide cough sound into overlapping blocks of size 30.

Step 5: Create a second matrix with dimension 6 × 5 using vector-to-matrix transformation.

Step 6: Use the pyrimidine pattern and signum function to generate 11 bits.

Step 7: Merge the generated bits (total 25 bits) from Steps 3 and 6.

Step 8: Divide these bits into left, middle and right groups.

(2) left(j)=bit(j), j∈{1,2,…,8}

(3) middle(k)=bit(k+8), k∈{1,2,…,9}

(4) right(j)=bit(j+17)

From Equations (2)–(4), left, middle and right bit groups contain 8, 9, and 8 bits, respectively.

Step 9: Create three map signals using the generated bit groups.

(5) $$m^{1}\left(i\right)=\overset{8}{\underset{j=1}{\sum }}left\left(j\right)*2^{j-1}$$

(6) $$m^{2}\left(i\right)=\overset{9}{\underset{k=1}{\sum }}middle\left(k\right)*2^{k-1}$$

(7) $$m^{3}\left(i\right)=\overset{8}{\underset{j=1}{\sum }}right\left(j\right)*2^{j-1}$$

where $m^{1},\;m^{2}$ and $m^{3}$ are the generated first, second, and third map sounds for feature generation. Histograms of these map sounds are extracted to obtain feature vectors. From Equations (5)–(9), these signals are coded with 8, 9, and 8 bits, respectively.

Step 10: Extract histograms of $m^{1},\;m^{2}$, and $m^{3}$. The lengths of the created histograms of $m^{1},\;m^{2}$, and $m^{3}$ are calculated as $2^{8},\;2^{9}$, and $2^{8}$, respectively.

Step 11: Merge the extracted histograms to obtain the feature vector of the DNA pattern.

(8) $$fv\left(a\right)=h^{1}\left(a\right),\;a\in \left\{1,2,\ldots ,256\right\}$$

(9) $$fv\left(g+256\right)=h^{2}\left(g\right),\;g\in \left\{1,2,\ldots ,512\right\}$$

(10) $$fv\left(a+768\right)=h^{3}\left(a\right)$$

where fv defines a feature vector with length 1024, and $h^{1},\;h^{2}$, and $h^{3}$ are histograms extracted using the $m^{1},\;m^{2}$, and $m^{3}$ map signals, respectively.

The eleven steps above define the DNA pattern-based feature generation. 1024 features are generated from each sound segment by deploying these steps.

#### 2.2.2. Feature Selection

**Figure 4:**
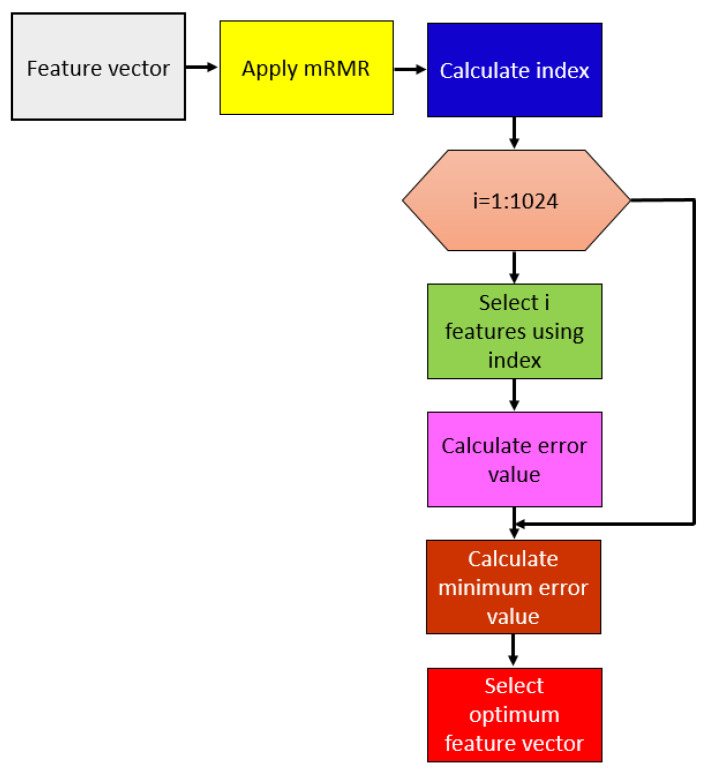
Steps involved in the selection of an optimal number of features using the ImRMR selector.

For automatic selection of the optimal number of generated features, we proposed an iterative version of the maximum relevance minimum redundancy selector (mRMR) [50], ImRMR, that incorporated an error calculator with kNN classifier. A schematic of the ImRMR selector is shown in Figure 4.

By deploying ImRMR, each of the 1024 features extracted by the DNA pattern is selected iteratively, and the kNN classifier employed to calculate the resultant error rates of the selected feature vector. The steps of the ImRMR used are detailed below.

Step 1: Apply mRMR and calculate 1024 index (id) values.

Step 2: Select features using the id that has been calculated in Step 1.

(11) $$sf^{i}\left(k,j\right)=fv\left(k,id\left(j\right)\right),\;i\in \left\{1,2,\ldots ,1024\right\},\;j\in \left\{1,2,\ldots ,i\right\},\;k\in \left\{1,2,\ldots ,2156\right\}$$

where $sf^{i}$ represents *i*th selected features, and k is the number of observations. Here, iterative feature selection is described.

Step 3: Calculate loss values of each feature vector selected using the kNN classifier with 10-fold cross-validation.

(12) $$\mu \left(i\right)=\mathrm{kNN}\left(sf^{i}\right)$$

In Equation (12), *μ* and kNN(.) represent the error value and the kNN classifier, respectively.

Step 4: Find the minimum loss value.

Step 5: Select optimal feature vector (last) using index (ind) of the minimum error value.

(13) last(k,j)=fv(k,id(j)), j∈{1,2,…,ind}, k∈{1,2,…,2156}

#### 2.2.3. Classification

A standard distance classifier (kNN) [19] was utilized for selecting the best and optimal number of feature vectors (it functioned as error value generator, see Section 2.2.2) as well as for calculating the classification results. Parameters of the kNN are: k was selected as one; distance parameter, Spearman; distance weight, equal; and standardize, true. Ten-fold cross-validation was chosen as the validation technique.

## 3. Results

### 3.1. Experimental Setup

The MATLAB (2020b) coding environment was used to develop the proposed DNA pattern- and ImRMR-based cough sound classification model. Systems configuration of the computer used were as follows:

Operating system: Window 10.1 professional,

RAM: 48 gigabytes,

CPU: Intel i9 9900 with 3.60 GHz cycling frequency,

Specifically, neither graphical core nor parallel processing was used to develop the model.

### 3.2. Cases

To evaluate the proposed model comprehensively, three distinct clinically relevant classification problems were defined based on the collected cough sound dataset:

Case 1: COVID-19 vs. healthy binary classification. 906 + 696 = 1602 observations were analyzed, and ImRMR was implemented to select 198 features.

Case 2: HF vs. healthy binary classification. 554 + 696 = 1250 observations were analyzed, and ImRMR selected 50 features.

Case 3: COVID-19 vs. HF vs. healthy multiclass classification. 906 + 554 + 696 = 2156 observations were analyzed, and ImRMR selected 895 features.

### 3.3. Results

**Figure 5:**
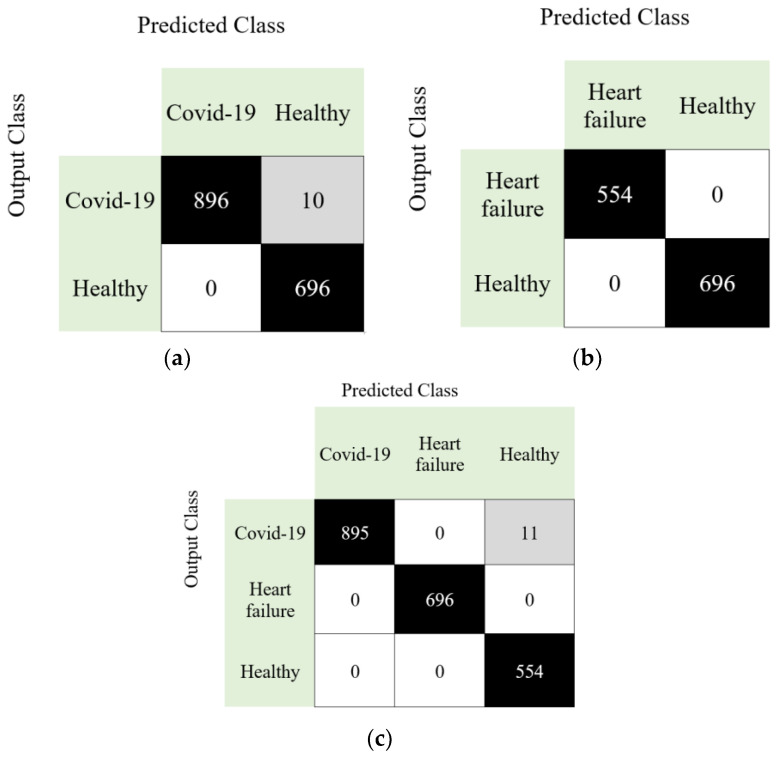
Confusion matrices obtained for various Cases (**a**) Case 1, (**b**) Case 2, (**c**) Case 3.

Standard performance metrics including accuracy, sensitivity, precision, F1-score, and geometric mean [51] were evaluated (see Table 1) and confusion matrices constructed (Figure 5) for all Cases. High classification accuracy rates of 99.38%, 100% and 99.49% were attained for Case 1, Case 2 and Case 3, respectively, with low rates of classification error.

The time burden (computational complexity) of the presented model was denoted using big O notation. The time complexity of the DNA pattern-based local feature generator function was O(n), where *n* was the length of the cough sound segment analyzed. ImRMR used both kNN and mRMR, and constituted the most complex phase of the model. Its time burden was $O\left(Mlnd^{2}\right)$, where M, l and d were the iteration number, length of the features, and number of observations, respectively. In the classification phase, kNN was used and the associated time complexity was O(nd).

## 4. Discussion

**Figure 6:**
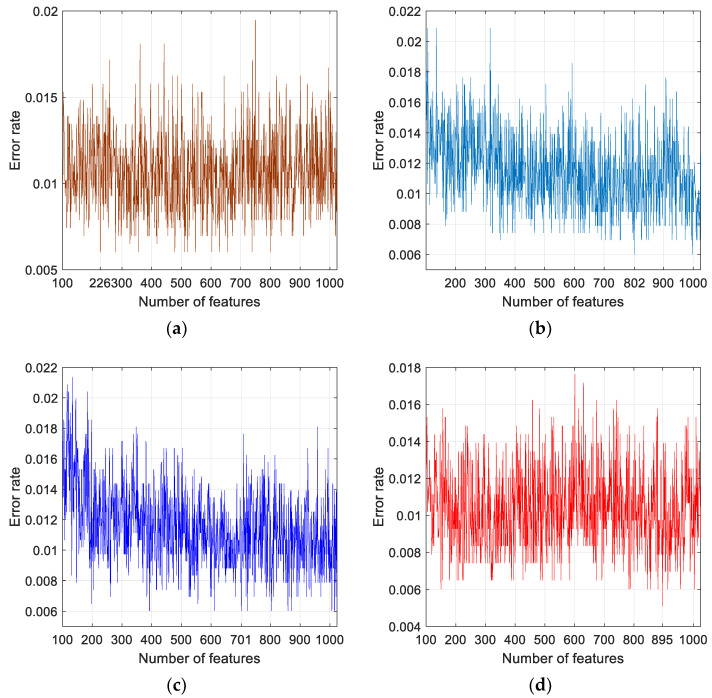
Plots of error rates versus number of features selected using: (**a**) iterative feature selectors IChi2, (**b**), INCA, (**c**), IRF, and (**d**) ImRMR implemented for Case 3.

Cough sound-based COVID-19 detection is an emerging field of research for both clinicians and machine learning experts. The prevalence and incidence of HF has been on the increase even before the onset of the COVID-19 pandemic, and is now often affected by a lack of access to routine medical care. The clinical presentations of both COVID-19 and HF can overlap, which underscores the need for the development of computer-aided diagnostic tools to support clinicians in triage and management. Both conditions can induce cough symptoms. Therefore, we collected cough sounds from COVID-19 and HF patients, as well as healthy subjects, to test the performance of our proposed DNA pattern- and ImRMR-based model. Our proposed model is able to classify three clinically relevant classification problems: COVID-19 vs. healthy; HF vs. healthy; and COVID-19 vs. HF vs. healthy. The model generated 1024 features from each one-second cough sound segment. An iterative feature selector is employed to select the most discriminative features. We presented the results obtained using ImRMR, iterative neighborhood component analysis (INCA), iterative ReliefF (IRF) and iterative Chi2 (IChi2) feature selectors. The plots of error rates versus number of features selected using these feature selectors implemented for Case 3 are shown in Figure 6.

**Figure 7:**
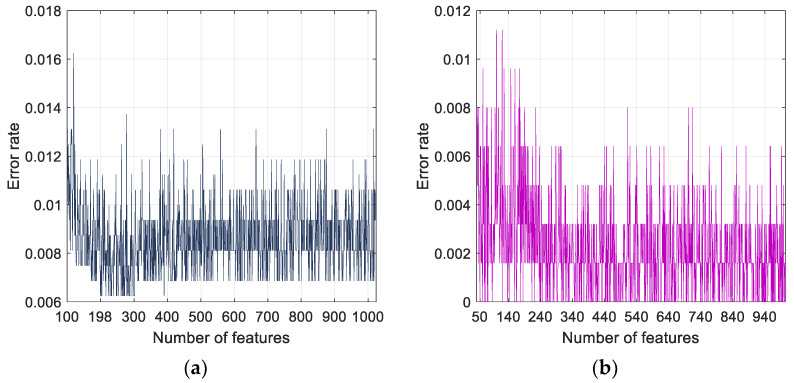
Plots of error rates versus number of features selected by ImRMR for (**a**) Case 1 and (**b**) Case 2.

It can be noted from Figure 6 that the number of features selected corresponding to least error rates for Case 3 classification using IChi2, INCA, IRF and ImRMR are 226, 802, 701, and 895, respectively. The minimum error rate of 0.0051 is obtained for ImRMR, 0.006 for IChi2, INCA, and IRF selectors. Application of ImRMR to Case 1 and Case 2 yielded minimum error rates of 0.0062 and 0 for 198 and 50 selected features, respectively (Figure 7). Overall, the model attained 99.38%, 100% and 99.49% accuracy rates for Case 1, Case 2 and Case 3 classifications, respectively.

**Figure 8:**
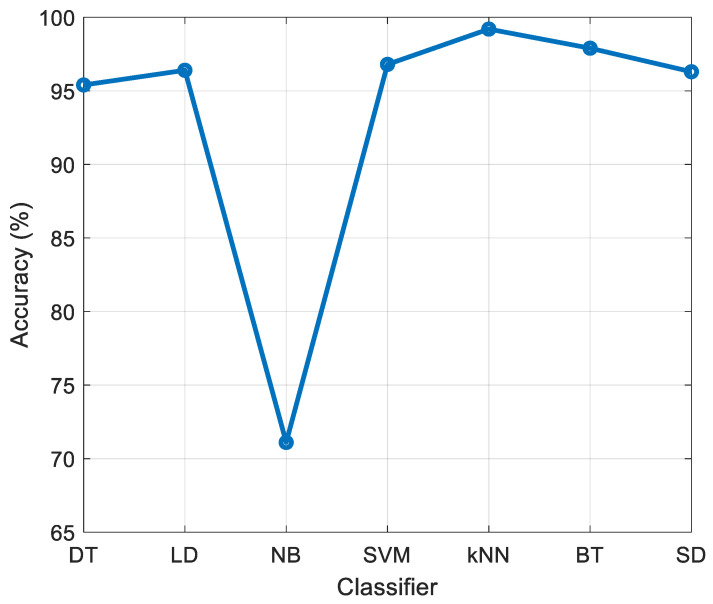
Classification accuracy (%) obtained for various classifiers using 1024 generated features.

The Standard kNN classifier is employed for calculating the error rate during the feature selection phase (see Section 2.2.3) in order to obtain classification results. We have used decision tree (DT) [52], linear discriminant (LD) [53], naïve Bayes (NB) [54], support vector machine (SVM) [55], kNN [19], bagged tree (BT) [56], and subspace discriminant (SD) [57] classifiers in addition to kNN for the classification tasks using 1024 features. It can be noted from Figure 8 that the best results are obtained using the kNN classifier. Therefore, kNN is selected both as the classifier and the error/loss value generator in the features selection phase.

The performance parameters (%) obtained for automated COVID-19 detection using cough sound signals is depicted in Table 2.

The benefits and disadvantages of our proposed DNA pattern-based method are given below.

The benefits are as follows.

- Developed a new cough sound dataset, which was collected from healthy subjects, and COVID-19 and HF patients.
- Presented a novel histogram-based feature generator inspired by DNA patterns. To the best our knowledge, this is the first work to automatically classify healthy subjects, HF and COVID-19 patients using cough sounds signals.
- Proposed a DNA pattern- and ImRMR-based model which attained greater than 99% accuracy for all (binary and multiclass) defined classification problems.
- Generated an automated model based on cough sounds that is accurate, economical, rapid, and computationally lightweight.
- The limitations of this work are given below:
- The system should be validated with a larger dataset prior to clinical application.
- Only a three-class system was used (normal, COVID-19 and HF).

**Figure 9:**
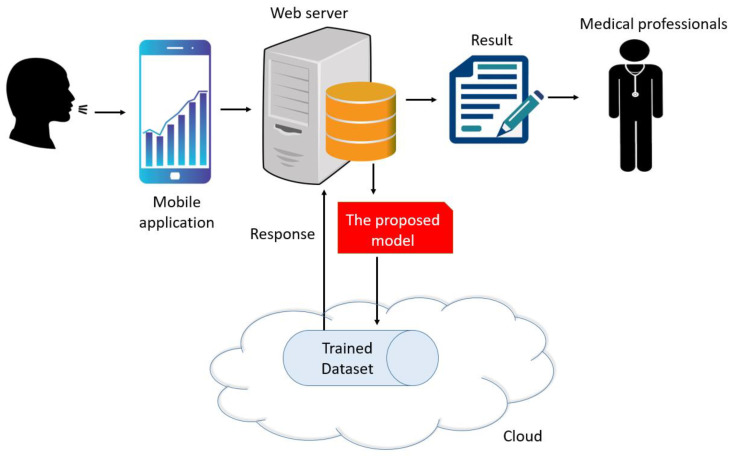
Snapshot of cloud-based cough detection via mobile application with cough sounds.

We have presented a histogram-based hand-modeled feature generation function using the DNA molecular pattern. New-generation deep learning models based on molecular shapes can be further studied to improve model performance. A snapshot of cloud-based cough detection via mobile application with cough sounds is presented in Figure 9.

## 5. Conclusions

This paper presents a new automated COVID-19 and HF failure detection model using cough sounds. This model extracts subtle features from a cough sound signal using a histogram-based feature generator with a chemical structure of DNA molecule. The proposed DNA patterns used for feature bit generation, combined with the ImRMR and kNN classifier, yielded an accuracy of 99.38%, 100%, and 99.49% for COVID-19 vs. healthy, HF vs. healthy, and COVID-19 vs. HF vs. healthy diagnoses, respectively. The model is accurate, economical and computationally lightweight. In the future, we intend to detect asthma in addition to the three classes currently used for cough sound signal analysis.

## Figures and Tables

**Table 1:** Model performance metrics (%) obtained for various Cases.

Case	Accuracy (%)	Sensitivity (%)	Precision (%)	F1-Score (%)	Geometric Mean (%)
Case 1	99.38	98.90	100	99.45	99.45
Case 2	100	100	100	100	100
Case 3	99.49	99.60	99.35	99.47	99.59

**Table 2:** Performance metrics (%) obtained for automated COVID-19 detection using cough sound signals.

Study	Method	Classifier	Dataset	Subjects/Samples	Results (%)
Brown et al. [58]	Mel-Frequency Cepstral Coefficients	Support vector machine	Collected data	23 COVID-19 with cough 29 No-covid19 with cough	AUC: 82.00 Pre: 80.00 Rec: 72.00
Wei et al. [59]	Convolution neural networks, Mel-frequency cepstral coefficients	Support vector machine	Collected data	64 COVID-19 40 Healthy 20 Bronchitis 20 Chronic pharyngitis 10 children with pertussis 39 Smoking subject	Sen: 98.70 Spe: 94.70 for COVID-19
Xia et al. [60]	Convolutional neural networks	Softmax	Collected data	330 COVID-19 688 Healthy	AUC: 74.00 Sen: 68.00 Spe: 69.00
Hassan et al. [61]	Recurrent neural network, long-short term memory	Recurrent neural network	Collected data	60 Healthy 20 COVID-19	Acc: 97.00 AUC: 97.40 F1: 97.90 Rec: 96.40 Pre:99.30
Pahar et al. [62]	Mel frequency cepstral coefficients, log energies, zero-crossing rate, kurtosis	Long short-term memory, sequential forward search	1. Coswara [63], 2. Sarcos [64] dataset	1. 1079 healthy 92 COVID-19 2. 13 COVID-19 negative 8 COVID-19 positive	Spe: 96.00 Sen: 91.00 Acc: 92.91 AUC: 93.75 for combined dataset
Schuller et al. [65]	Deep spectrum, autoencoders	Convolutional neural networks	Cambridge COVID-19 sound database [58,66]	119 COVID-19 606 No-COVID-19	UAR: 73.90
Andreu-Perez et al. [67]	Empirical mode decomposition, convolutional neural networks	Artificial neural network	Collected data	2339 COVID-19 positive 6041 COVID-19 negative	AUC: 66.41 Pre: 76.04 Sen: 76.64 Spe: 67.00
Chowdhury et al. [68]	Convolutional neural networks	Convolutional neural networks	Coswara [63], Cambridge [58], CoughVid [69] dataset.	582 healthy 141 COVID-19 patients	Acc: 95.86 Pre: 95.84 Sen: 95.86 F1: 95.84 Spe: 93.43
Maleki [70]	Mel frequency cepstral coefficients, Sequential forward selection	Euclidean k-nearest neighbors	Combined dataset (Virufy COVID-19 open cough data set [71], NoCoCoDa [72])	48 COVID-19 positive 73 COVID-19 negative	Acc: 98.33 F1: 97.99 AUC: 98.60 Sen: 100.0 for Non-COVID-19 Sen: 97.20 for COVID-19
Mouawad et al. [73]	Mel frequency cepstral coefficients, recurrence quantification analysis	Weighted XGBoost	Collected data	1895 healthy 32 sick samples	Acc: 97.00 F1: 62.00 AUC: 84.00
Our method	DNA pattern	k-nearest neighbors	Collected data	247 healthy 241 COVID-19 244 heart failure	Acc: 99.38 Sen: 98.90 Pre: 100.0 F1: 99.45 Gm: 99.45 for Case 1
					Acc: 100.0 Sen: 100.0 Pre: 100.0 F1: 100.0 Gm: 100.0 for Case 2
					Acc: 99.49 Sen: 99.60 Pre: 99.35 F1: 99.47 Gm: 99.59 for Case 3

AUC: Area under the ROC curve, Acc: Accuracy, Sen: Sensitivity, Spe: Specificity, Pre: Precision, F1: F1-Score, Gm: Geometric mean, Rec: Recall.

## Data Availability

The data presented in this study are available on request from the corresponding author. The data are not publicly available due to restrictions regarding the Ethical Committee Institution.

## References

[1] Hussain E., Hasan M., Rahman M.A., Lee I., Tamanna T., Parvez M.Z. (2021). CoroDet: A deep learning based classification for COVID-19 detection using chest X-ray images. Chaos Solitons Fractals.

[2] Okoshi H., Suzuki H., Nakano A., Hamada A., Miyamoto T., Yamasawa F. (2020). A Guide to Novel Coronavirus (COVID-19) Infection Control for Businesses. J. Occup. Health.

[3] Sheffi Y. (2020). The New (Ab) Normal: Reshaping Business and Supply Chain Strategy Beyond COVID-19.

[4] Kanne J.P., Bai H., Bernheim A., Chung M., Haramati L.B., Kallmes D.F., Little B.P., Rubin G.D., Sverzellati N. (2021). COVID-19 imaging: What we know now and what remains unknown. Radiology.

[5] Hall G., Laddu D.R., Phillips S.A., Lavie C.J., Arena R. (2020). A tale of two pandemics: How will COVID-19 and global trends in physical inactivity and sedentary behavior affect one another?. Prog. Cardiovasc. Dis..

[6] Megahed N.A., Ghoneim E.M. (2020). Antivirus-built environment: Lessons learned from COVID-19 pandemic. Sustain. Cities Soc..

[7] Agarwal S., Punn N.S., Sonbhadra S.K., Nagabhushan P., Pandian K., Saxena P. (2020). Unleashing the power of disruptive and emerging technologies amid COVID 2019: A detailed review. arXiv.

[8] Shchendrygina A., Nagel E., Puntmann V.O., Valbuena-Lopez S. (2021). COVID-19 myocarditis and prospective heart failure burden. Expert Rev. Cardiovasc. Ther..

[9] Pahuja G., Nagabhushan T. (2021). A comparative study of existing machine learning approaches for parkinson’s disease detection. IETE J. Res..

[10] Miah Y., Prima C.N.E., Seema S.J., Mahmud M., Kaiser M.S. (2021). Performance comparison of machine learning techniques in identifying dementia from open access clinical datasets. Advances on Smart and Soft Computing.

[11] Aslan M.F., Unlersen M.F., Sabanci K., Durdu A. (2021). CNN-based transfer learning–BiLSTM network: A novel approach for COVID-19 infection detection. Appl. Soft Comput..

[12] Gupta A., Gupta R., Garg N. (2021). An efficient approach for classifying chest X-ray images using different embedder with different activation functions in CNN. J. Interdiscip. Math..

[13] Nazari S., Fallah M., Kazemipoor H., Salehipour A. (2018). A fuzzy inference-fuzzy analytic hierarchy process-based clinical decision support system for diagnosis of heart diseases. Expert Syst. Appl..

[14] Maghdid H.S., Asaad A.T., Ghafoor K.Z., Sadiq A.S., Khan M.K. (2020). Diagnosing COVID-19 pneumonia from X-ray and CT images using deep learning and transfer learning algorithms. arXiv.

[15] Huang Q. (2018). Occupancy-driven energy-efficient buildings using audio processing with background sound cancellation. Buildings.

[16] Zheng J., Lu C., Hao C., Chen D., Guo D. (2020). Improving the generalization ability of deep neural networks for cross-domain visual recognition. IEEE Trans. Cogn. Dev. Syst..

[17] Tao H., Wu T., Aldeghi M., Wu T.C., Aspuru-Guzik A., Kumacheva E. (2021). Nanoparticle synthesis assisted by machine learning. Nat. Rev. Mater..

[18] Tuncer T., Dogan S., Tan R.-S., Acharya U.R. (2021). Application of Petersen graph pattern technique for automated detection of heart valve diseases with PCG signals. Inf. Sci..

[19] Maillo J., Ramírez S., Triguero I., Herrera F. (2017). kNN-IS: An Iterative Spark-based design of the k-Nearest Neighbors classifier for big data. Knowl. Based Syst..

[20] Masetic Z., Subasi A. (2016). Congestive heart failure detection using random forest classifier. Comput. Methods Programs Biomed..

[21] Goldberger A.L., Amaral L.A., Glass L., Hausdorff J.M., Ivanov P.C., Mark R.G., Mietus J.E., Moody G.B., Peng C.-K., Stanley H.E. (2000). PhysioBank, PhysioToolkit, and PhysioNet: Components of a new research resource for complex physiologic signals. Circulation.

[22] Ralf-Dieter B. The PTB Diagnostic ECG Database. http://www.physionet.org/physiobank/database/ptbdb/.

[23] Baim D.S., Colucci W.S., Monrad E.S., Smith H.S., Wright R.F., Lanoue A., Gauthier D.F., Ransil B.J., Grossman W., Braunwald E. (1986). Survival of patients with severe congestive heart failure treated with oral milrinone. J. Am. Coll. Cardiol..

[24] Tripathy R.K., Paternina M.R., Arrieta J.G., Zamora-Méndez A., Naik G.R. (2019). Automated detection of congestive heart failure from electrocardiogram signal using Stockwell transform and hybrid classification scheme. Comput. Methods Programs Biomed..

[25] Moody G.B., Mark R.G. (2001). The impact of the MIT-BIH arrhythmia database. IEEE Eng. Med. Biol. Mag..

[26] Porumb M., Iadanza E., Massaro S., Pecchia L. (2020). A convolutional neural network approach to detect congestive heart failure. Biomed. Signal Process. Control.

[27] Abbas A., Abdelsamea M.M., Gaber M.M. (2021). Classification of COVID-19 in chest X-ray images using DeTraC deep convolutional neural network. Appl. Intell..

[28] Candemir S., Jaeger S., Palaniappan K., Musco J.P., Singh R.K., Xue Z., Karargyris A., Antani S., Thoma G., McDonald C.J. (2013). Lung segmentation in chest radiographs using anatomical atlases with nonrigid registration. IEEE Trans. Med. Imaging.

[29] Jaeger S., Karargyris A., Candemir S., Folio L., Siegelman J., Callaghan F., Xue Z., Palaniappan K., Singh R.K., Antani S. (2013). Automatic tuberculosis screening using chest radiographs. IEEE Trans. Med. Imaging.

[30] Jaiswal A., Gianchandani N., Singh D., Kumar V., Kaur M. (2020). Classification of the COVID-19 infected patients using DenseNet201 based deep transfer learning. J. Biomol. Struct. Dyn..

[31] SARS-COV-2 Ct-Scan Dataset. www.kaggle.com/plameneduardo/sarscov2-ctscan-dataset.

[32] Singh D., Kumar V., Kaur M. (2020). Classification of COVID-19 patients from chest CT images using multi-objective differential evolution–based convolutional neural networks. Eur. J. Clin. Microbiol. Infect. Dis..

[33] Horry M.J., Chakraborty S., Paul M., Ulhaq A., Pradhan B., Saha M., Shukla N. (2020). COVID-19 detection through transfer learning using multimodal imaging data. IEEE Access.

[34] Cohen J.P., Morrison P., Dao L., Roth K., Duong T.Q., Ghassemi M. (2020). COVID-19 image data collection: Prospective predictions are the future. arXiv.

[35] Wang X., Peng Y., Lu L., Lu Z., Bagheri M., Summers R.M. (2017). Chestx-ray8: Hospital-scale chest x-ray database and benchmarks on weakly-supervised classification and localization of common thorax diseases. arXiv.

[36] Yang X., He X., Zhao J., Zhang Y., Zhang S., Xie P. (2020). Covid-ct-dataset: A ct scan dataset about COVID-19. arXiv.

[37] Born J., Brändle G., Cossio M., Disdier M., Goulet J., Roulin J., Wiedemann N. (2020). POCOVID-Net: Automatic detection of COVID-19 from a new lung ultrasound imaging dataset (POCUS). arXiv.

[38] Simonyan K., Zisserman A. (2015). Very deep convolutional networks for large-scale image recognition. arXiv.

[39] Chollet F. (2017). Xception: Deep learning with depthwise separable convolutions. arXiv.

[40] Szegedy C., Ioffe S., Vanhoucke V., Alemi A. (2016). Inception-v4, inception-resnet and the impact of residual connections on learning. arXiv.

[41] Szegedy C., Vanhoucke V., Ioffe S., Shlens J., Wojna Z. (2015). Rethinking the inception architecture for computer vision. arXiv.

[42] Zoph B., Vasudevan V., Shlens J., Le Q.V. (2018). Learning transferable architectures for scalable image recognition. arXiv.

[43] Huang G., Liu Z., Van Der Maaten L., Weinberger K.Q. (2018). Densely connected convolutional networks. arXiv.

[44] He K., Zhang X., Ren S., Sun J. (2015). Deep residual learning for image recognition. arXiv.

[45] Zebin T., Rezvy S. (2021). COVID-19 detection and disease progression visualization: Deep learning on chest X-rays for classification and coarse localization. Appl. Intell..

[46] Wardhani N.W.S., Rochayani M.Y., Iriany A., Sulistyono A.D., Lestantyo P. Cross-validation metrics for evaluating classification performance on imbalanced data. Proceedings of the 2019 International Conference on Computer, Control, Informatics and its Applications (IC3INA).

[47] Krithika L., Priya G.L. (2021). Graph based feature extraction and hybrid classification approach for facial expression recognition. J. Ambient Intell. Humaniz. Comput..

[48] Yu X., Wang S.-H., Zhang Y.-D. (2021). CGNet: A graph-knowledge embedded convolutional neural network for detection of pneumonia. Inf. Process. Manag..

[49] Gupta A., Matta P., Pant B. (2021). Graph neural network: Current state of Art, challenges and applications. Mater. Today Proc..

[50] Unler A., Murat A., Chinnam R.B. (2011). mr2PSO: A maximum relevance minimum redundancy feature selection method based on swarm intelligence for support vector machine classification. Inf. Sci..

[51] Tuncer T., Dogan S., Pławiak P., Acharya U.R. (2019). Automated arrhythmia detection using novel hexadecimal local pattern and multilevel wavelet transform with ECG signals. Knowl. Based Syst..

[52] Safavian S.R., Landgrebe D. (1991). A survey of decision tree classifier methodology. IEEE Trans. Syst. Man Cybern..

[53] Zhao W., Chellappa R., Nandhakumar N. Empirical performance analysis of linear discriminant classifiers. Proceedings of the 1998 IEEE Computer Society Conference on Computer Vision and Pattern Recognition (Cat. No. 98CB36231).

[54] Rish I. An empirical study of the naive Bayes classifier. Proceedings of the IJCAI 2001 Workshop on Empirical Methods in Artificial Intelligence.

[55] Vapnik V. (2013). The nature of Statistical Learning Theory.

[56] Mishra P.K., Yadav A., Pazoki M. (2018). A novel fault classification scheme for series capacitor compensated transmission line based on bagged tree ensemble classifier. IEEE Access.

[57] Ashour A.S., Guo Y., Hawas A.R., Xu G. (2018). Ensemble of subspace discriminant classifiers for schistosomal liver fibrosis staging in mice microscopic images. Health Inf. Sci. Syst..

[58] Brown C., Chauhan J., Grammenos A., Han J., Hasthanasombat A., Spathis D., Xia T., Cicuta P., Mascolo C. Exploring automatic diagnosis of COVID-19 from crowdsourced respiratory sound data. Proceedings of the 26th ACM SIGKDD International Conference on Knowledge Discovery & Data Mining.

[59] Wei W., Wang J., Ma J., Cheng N., Xiao J. (2020). A Real-time Robot-based Auxiliary System for Risk Evaluation of COVID-19 Infection. arXiv.

[60] Xia T., Han J., Qendro L., Dang T., Mascolo C. (2021). Uncertainty-Aware COVID-19 Detection from Imbalanced Sound Data. arXiv.

[61] Hassan A., Shahin I., Alsabek M.B. COVID-19 detection system using recurrent neural networks. Proceedings of the 2020 International Conference on Communications, Computing, Cybersecurity, and Informatics (CCCI).

[62] Pahar M., Klopper M., Warren R., Niesler T. (2020). COVID-19 Cough Classification using Machine Learning and Global Smartphone Recordings. arXiv.

[63] Sharma N., Krishnan P., Kumar R., Ramoji S., Chetupalli S.R., Ghosh P.K., Ganapathy S. (2020). Coswara—A Database of Breathing, Cough, and Voice Sounds for COVID-19 Diagnosis. arXiv.

[64] Sarcos. https://coughtest.online.

[65] Schuller B.W., Batliner A., Bergler C., Mascolo C., Han J., Lefter I., Kaya H., Amiriparian S., Baird A., Stappen L. (2021). The INTERSPEECH 2021 Computational Paralinguistics Challenge: COVID-19 cough, COVID-19 speech, escalation & primates. arXiv.

[66] Han J., Brown C., Chauhan J., Grammenos A., Hasthanasombat A., Spathis D., Xia T., Cicuta P., Mascolo C. (2021). Exploring Automatic COVID-19 Diagnosis via voice and symptoms from Crowdsourced Data. arXiv.

[67] Andreu-Perez J., Pérez-Espinosa H., Timonet E., Kiani M., Giron-Perez M.I., Benitez-Trinidad A.B., Jarchi D., Rosales A., Gkatzoulis N., Reyes-Galaviz O.F. (2021). A Generic Deep Learning Based Cough Analysis System from Clinically Validated Samples for Point-of-Need COVID-19 Test and Severity Levels. IEEE Trans. Serv. Comput..

[68] Chowdhury M.E., Ibtehaz N., Rahman T., Mekki Y.M.S., Qibalwey Y., Mahmud S., Ezeddin M., Zughaier S., Al-Maadeed S.A.S. (2021). QUCoughScope: An Artificially Intelligent Mobile Application to Detect Asymptomatic COVID-19 Patients using Cough and Breathing Sounds. arXiv.

[69] Orlandic L., Teijeiro T., Atienza D. (2020). The COUGHVID crowdsourcing dataset: A corpus for the study of large-scale cough analysis algorithms. arXiv.

[70] Maleki M. (2021). Diagnosis of COVID-19 and Non-COVID-19 Patients by Classifying Only a Single Cough Sound. arXiv.

[71] Chaudhari G., Jiang X., Fakhry A., Han A., Xiao J., Shen S., Khanzada A. (2020). Virufy: Global Applicability of Crowdsourced and Clinical Datasets for AI Detection of COVID-19 from Cough. arXiv.

[72] Cohen-McFarlane M., Goubran R., Knoefel F. (2020). Novel coronavirus cough database: Nococoda. IEEE Access.

[73] Mouawad P., Dubnov T., Dubnov S. (2021). Robust Detection of COVID-19 in Cough Sounds: Using Recurrence Dynamics and Variable Markov Model. SN Comput. Sci..

